# *Stenotrophomonas bentonitica* sp. nov., isolated from bentonite formations

**DOI:** 10.1099/ijsem.0.002016

**Published:** 2017-08-18

**Authors:** Iván Sánchez-Castro, Miguel Angel Ruiz-Fresneda, Mohammed Bakkali, Peter Kämpfer, Stefanie P. Glaeser, Hans Jürgen Busse, Margarita López-Fernández, Pablo Martínez-Rodríguez, Mohamed Larbi Merroun

**Affiliations:** ^1^​ Departamento de Microbiología, Campus de Fuentenueva, Universidad de Granada, 18071 Granada, Spain; ^2^​ Departamento de Genética, Campus de Fuentenueva, Universidad de Granada, 18071 Granada, Spain; ^3^​ Institut für Angewandte Mikrobiologie, Justus-Liebig-Universität Giessen, D-35392 Giessen, Germany; ^4^​ Institut für Mikrobiologie, Veterinärmedizinische Universität Wien, A-1210 Wien, Austria; ^†^​ Present address: Centre for Ecology and Evolution in Microbial Model Systems (EEMiS), Linnaeus University, Kalmar, Sweden.

**Keywords:** *Stenotrophomonas*, BII-R7^T^, bentonite, ANI value

## Abstract

A Gram-stain negative, rod-shaped, aerobic bacterial strain, BII-R7^T^, was isolated during a study targeting the culture-dependent microbial diversity occurring in bentonite formations from southern Spain. Comparative 16S rRNA gene sequence analysis showed that BII-R7^T^ represented a member of the genus *Stenotrophomonas* (class *Gammaproteobacteria*), and was related most closely to *Stenotrophomonas rhizophila* e-p10^T^ (99.2 % sequence similarity), followed by *Stenotrophomonas pavanii* ICB 89^T^ (98.5 %), *Stenotrophomonas maltophilia* IAM 12423^T^, *Stenotrophomonas chelatiphaga* LPM-5^T^ and *Stenotrophomonas tumulicola* T5916-2-1b^T^ (all 98.3 %). Pairwise sequence similarities to all other type strains of species of the genus *Stenotrophomonas* were below 98 %. Genome-based calculations (orthologous average nucleotide identity, original average nucleotide identity, genome-to-genome distance and DNA G+C percentage) indicated clearly that the isolate represents a novel species within this genus. Different phenotypic analyses, such as the detection of a quinone system composed of the major compound ubiquinone Q-8 and a fatty acid profile with iso-C_15 : 0_ and anteiso-C_15 : 0_ as major components, supported this finding at the same time as contributing to a comprehensive characterization of BII-R7^T^. Based on this polyphasic approach comprising phenotypic and genotypic/molecular characterization, BII-R7^T^ can be differentiated clearly from its phylogenetic neighbours, establishing a novel species for which the name *Stenotrophomonas bentonitica* sp. nov. is proposed with BII-R7^T^ as the type strain (=LMG 29893^T^=CECT 9180^T^=DSM 103927^T^).

A large number of microbial strains were isolated in a study targeting the culture-dependent microbial diversity occurring in bentonite formations from southern Spain. This investigation aimed at understanding the effects of microbial processes on the performance of this type of material for deep geological disposal of nuclear wastes [1]. By using standard dilution plating technique on different culture media, including oligotrophic R2A medium [2], Luria–Bertani (LB) medium [3] and nutrient broth (NB), 32 microbial isolates (31 bacterial strains and 1 fungal strain) were isolated and characterized. The strain BII-R7^T^, affiliated to the genus *Stenotrophomonas* (family *Xanthomonadaceae*, order *Xanthomonadales*, class *Gammaproteobacteria*) on the basis of 16S rRNA gene sequence divergence [1], was further investigated.

Species of the genus *Stenotrophomonas* possess an important ecological role in the element cycle in nature [4] and various potential biotechnological applications, for example as bioremediation agents [5–9], and are considered as potential plant growth-promoting and biocontrol organisms [10, 11], becoming a widely studied group. In this sense, the bacterial strain BII-R7^T^ showed high uranium and selenium tolerance, being able to grow up to 6 mM U [1] and 100 mM Se (Ruiz‐Fresneda MA, Gómez‐Bolívar J, Sánchez‐Castro I, Merroun ML, unpublished data). The taxonomy of the genus *Stenotrophomonas* has been subject to considerable revision over recent years. Originally, this genus was proposed when the species *Xanthomonas maltophilia* was reclassified as *Stenotrophomonas maltophilia* [12], and subsequently accommodated in the class *Gammaproteobacteria* [13]. At the time of writing, the genus *Stenotrophomonas* comprises 13 species with validly published names isolated from a large range of natural and artificial environments and geographical regions including *S. maltophilia* [12], *Stenotrophomonas nitritireducens* [14], *Stenotrophomonas acidaminiphila* [15], *Stenotrophomonas rhizophila* [16], *Stenotrophomonas koreensis* [17], *Stenotrophomonas humi* [18], *Stenotrophomonas terrae* [18], *Stenotrophomonas chelatiphaga* [19], *Stenotrophomonas ginsengisoli* [20], *Stenotrophomonas panacihumi* [21], *Stenotrophomonas daejeonensis* [22], *Stenotrophomonas pavanii* [23] and *Stenotrophomonas tumulicola* [24].

So far, species within the genus *Stenotrophomonas* have been described as being Gram-stain-negative, non-endospore-forming, rod-shaped, resistant to certain antibiotics and metals and catalase-positive. Moreover, the predominant cellular fatty acid component is iso-C_15 : 0_ and the DNA G+C content is between 64.0 and 69.1 mol% [12, 14–24]. BII-R7^T^ displays all these common characteristics. This fact, together with 16S rRNA gene sequencing [1], confirms that BII-R7^T^ represents a member of the genus *Stenotrophomonas*. However, considering that the 16S rRNA gene is not discriminative enough to classify certain strains at species level within this genus [25], a polyphasic approach comprising phenotypic and genotypic/molecular assays was employed to study the relationship of BII-R7^T^ with species of the genus *Stenotrophomonas*.

As a preliminary molecular characterization, almost the complete 16S rRNA gene was re-sequenced according to previously described methods [1]. The resulting sequence (1385 bp; GenBank accession number LT622838) was almost identical to the original sequence (1454 bp; GenBank accession number HG800055) [1] when aligned with the silva Incremental Aligner (sina; v1.2.11) [26]. For detailed phylogenetic placement of BII-R7^T^, its 16S rRNA gene sequence was aligned with the silva Incremental Aligner and implemented into the ‘All Species Living Tree Project’ (LTP) [27] using the arb software package release 5.2 [28] for analysis. Additionally, sequences not included in the LTP database were obtained from GenBank (http://www.ncbi.nlm.nih.gov/) and added to the database. Finally, the sequence alignment was checked manually. Pairwise sequence similarities were determined in arb using the arb neighbour-joining tool without the application of an evolutionary model. Phylogenetic trees were reconstructed with the maximum-likelihood method using RAxML version 7.04 [29] with General Time Reversible-GAMMA (GTR-GAMMA) and rapid bootstrap analysis, the maximum-parsimony method using dnapars v 3.6 [30], and the neighbour-joining methods using arb neighbour-joining and the Jukes–Cantor correction [31]. Independent of the applied treeing method, BII-R7^T^ was placed within the genus *Stenotrophomonas* and formed a distinct cluster with the type strain of *S. rhizophila* but not with any other type strains of species of the genus *Stenotrophomonas* (Fig. 1). The clustering of the two strains was always supported by high bootstrap values. The two strains shared 98.8 % 16S rRNA gene sequence similarity with each other, based on the blast analysis in EzTaxon [32], and 99.2 % sequence similarities based on the analysis in arb. Pairwise 16S rRNA gene sequence similarities (calculated in arb) between BII-R7^T^ and other type strains of species of the genus *Stenotrophomonas* were between 96.6 and 98.5 % 16S rRNA gene sequence similarity (Table S1, available in the online Supplementary Material). Sequence similarities of BII-R7^T^ to the type strains of *S. pavanii* (98.5 %), *S. maltophilia*, *S. chelatiphaga*, and *S. tumulicola* (all 98.3 %) were above 98 %. All other sequence similarities were below 98 %. Phylogenetic analysis also included 16S rRNA gene sequences of strains misclassified as representing species of the genus *Pseudomonas* (Fig. 1); pairwise 16S rRNA gene sequence similarities of BII-R7^T^ to those strains were always below 98.0 % (Table S1).

**Fig. 1.**
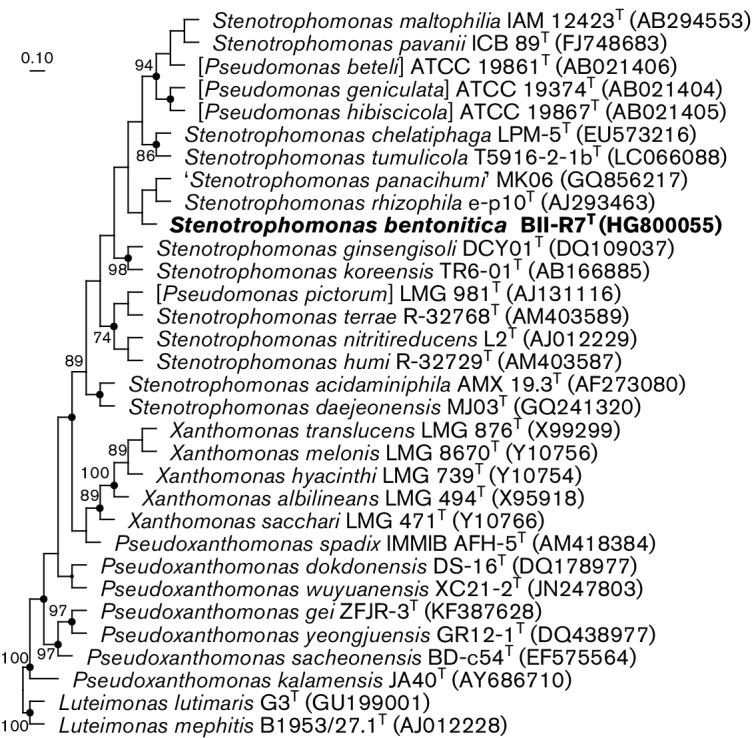
Maximum-likelihood phylogenetic tree based on nearly full-length 16S rRNA gene sequence of BII-R7^T^ and type strains of species of the genus *Stenotrophomonas* as well as species wrongly classified as members of the genus *Pseudomonas* and other related taxa. The 16S rRNA gene sequences of the type strains of *Luteimonas mephitis* and *Luteimonas lutimaris* were used as outgroups. Analysis based on 16S rRNA gene sequences between gene termini 67 and 1448 (according to the *Escherichia coli* numbering [54]). Sequence accession numbers are given in parentheses, including the BII-R7^T^ sequence obtained originally in this work. Circles at branch points represent those branch points which were also present in the phylogenetic trees obtained with other treeing methods. Bootstrap values greater than 70 % are shown at branch points (percentages of 100 re-samplings). Bar, 0.1 substitutions per sequence position.

For more detailed phylogenetic analysis, nucleotide sequences of the *gyrB* region 1 and *gyrB* region 2 were analyzed according to the methods of Svensson-Stadler *et al*. [25] including the *gyrB* gene sequences of all type strains of species of the genus *Stenotrophomonas*. The partial gene sequences of BII-R7^T^ were obtained from the genome sequence generated for the strain (see below). Reference sequences were taken either from Svensson-Stadler *et al.* [25], Handa *et al.* [24] or from published type strain genomes. The analysis was performed in mega 7 version 7.0 [33]. The nucleotide sequences alignment was obtained by the alignment of respective amino acid sequences using ClustalW implemented in mega7 and the phylogenetic trees were reconstructed using the maximum-likelihood method and the GTR method [34]. The final tree based on 100 replications (bootstrap analysis). Pairwise sequence similarities were calculated based on the determination of *p*-distances using mega7. In the obtained phylogenetic trees, BII-R7^T^ clustered either with the type strain of *S. chelatiphaga* and subsequently with *S. rhizophila* (*gyrB*, region 1, Fig. 2a) or directly with the type strain of *S. rhizophila* (*gyrB*, region 2, Fig. 2b). Pairwise sequence similarities for both partial gene sequences for BII-R7^T^ to other species of the genus *Stenotrophomonas* were all below 90 % (Tables S2 and S3) which supported the assignment of BII-R7^T^ to a novel species according to Svensson-Stadler *et al*. [25].

**Fig. 2.**
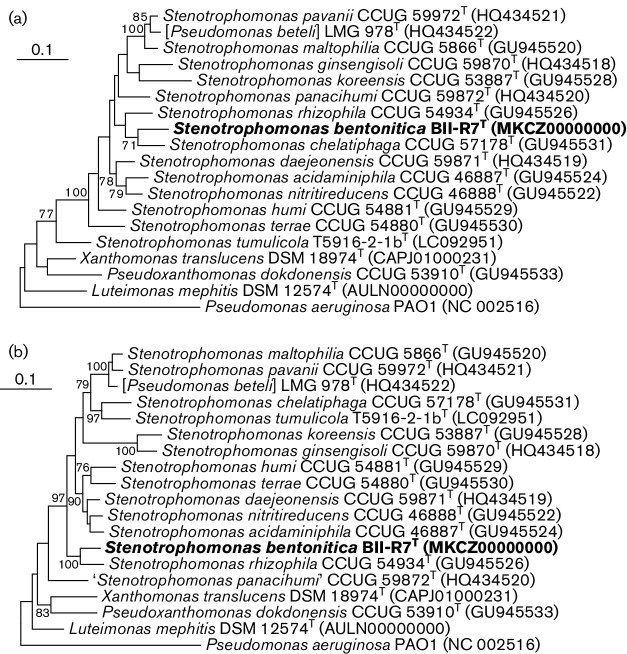
Maximum-likelihood phylogenetic tree based on partial *gyrB* gene sequence (a: region 1, b: region 2, according to Svensson-Stadler *et al*. [25]) of BII-R7^T^ and type strains of species of the genus *Stenotrophomonas* as well as species wrongly classified as members of the genus *Pseudomonas* and other related taxa. The *gyrB* gene sequences of *Luteimonas mephitis* and *Pseudomonas aeruginosa* were used as outgroups. Sequence accession numbers are given in parentheses. The trees were reconstructed in mega7 with the maximum-likelihood method using the GTR model and 100 replications. Bootstrap values greater than 70 % are shown at branch points (percentages of 100 re-samplings). Bars, 0.1 substitutions per sequence position.

Sequencing of the draft genome of BII-R7^T^ (GenBank accession number MKCZ00000000) allowed genomic analyses which clearly separated this strain from established species within the genus *Stenotrophomonas* and four other strains misclassified as representing members of the genus *Pseudomonas*. Reference genome sequences corresponding to type strains from all other species belonging to these genera were obtained from public databases [35]. Analysis of average nucleotide identity (ANI) with only the orthologous genes (Ortho-ANI) [36] produced values below the proposed 95–96 % threshold for the species boundary [37] between BII-R7^T^ and reference genomes (Table 1). Based on this algorithm, the most closely related species with a validly published name was *S. rhizophila* DSM 14405^T^ with an Ortho-ANI value of 85.5 %. Moreover, as previously observed when using 16S rRNA gene sequence analysis,*S. pavanii* and *S. maltophilia* (together with *Pseudomonas geniculata*, *P. beteli* and *P. hibiscicola*, considered as synonyms of *S. maltophilia* [38]) followed *S. rhizophila* as most closely related species to BII-R7^T^ with Ortho-ANI values higher than 81.6 %. In this case, *S. tumulicola* was not considered since its genome was not available at the time. Similar indices calculated by different methods (original ANI value by Ortho-ANI software, ANI value by *EzBioCloud* website (http://www.ezbiocloud.net/tools/ani) based on algorithm published by Goris *et al.* [39] and ANI value obtained through the *Kostas lab* website (http://enve-omics.ce.gatech.edu/ani/) supported these results consistently (Table 1). The digital DNA–DNA hybridizations (dDDH) were determined online at http://ggdc.dsmz.de/distcalc2.php using the Genome-to-Genome Distance Calculation (GGDC) version 2.0 as described in Meier-Kolthoff *et al.* [40] (Table 1). These calculations produced *in silico* DNA–DNA hybridization values well below the 70 %, threshold to delimit a bacterial species [41, 42]. BII-R7^T^ and *S. rhizophila* were found to have a dDDH value of 29.9 % [identities/high-scoring segment pair (HSP) length formula], with a probability of equal to or above 70 % DDH of 0.1 %. All other comparisons resulted in lower dDDH values (Table 1). DNA G+C contents were calculated *in-silico* in all cases (Table 1). In the case of BII-R7^T^ it was 66.4 mol%, whereas those for the type strains of the closest relatives *S. rhizophila* and *S. pavanii* were 67.3 mol% and 66.9 mol%.

**Table 1.** Genome-based comparisons of BII-R7^T^ and other type strains of members of the genus *Stenotrophomonas* and *Pseudomonas* (misclassified) retrieved from Patil *et al.* [35]

Reference strain	Ortho-ANI % (OAT)	Original ANI % (OrAT)	ANI % (EzBioCloud)	ANI calculation (Kostas Lab)	GGDC distance (DSMZ)*	mol% G+C (BII-R7^T^=66.49 %)
*Stenotrophomonas maltophilia* ATCC 13637^T^	81.6	80.8	80.9	83.3 (82.9–83.0)†	25.0	66.1
*Stenotrophomonas rhizophila* DSM 14405^T^	85.5	85.0	85.1	86.0 (85.7–85.7)	29.9	67.3
*Stenotrophomonas chelatiphaga* DSM 21508^T^	81.2	80.5	80.5	82.7 (82.4–82.4)	24.3	66.5
*Stenotrophomonas acidaminiphila* JCM 13310^T^	81.0	80.3	80.3	82.5 (82.1–82.2)	24.3	68.0
*Stenotrophomonas daejeonensis* JCM 16244^T^	81.0	80.1	80.1	82.2 (81.9–81.9)	24.1	67.8
*Stenotrophomonas ginsengisoli* DSM 24757^T^	76.7	75.9	76.0	79.6 (79.4–79.5)	20.8	64.4
*Stenotrophomonas humi* DSM 18929^T^	79.0	78.2	78.3	81.2 (80.8–80.8)	22.7	63.4
*Stenotrophomonas koreensis* DSM 17805^T^	76.7	75.8	75.9	79.8 (79.6–79.5)	20.8	65.5
*Stenotrophomonas nitritireducens* DSM 12575^T^	81.1	80.4	80.5	83.1 (82.5–82.5)	24.5	66.0
‘*Stenotrophomonas panacihumi*’ JCM 16536	79.1	78.3	78.3	81.2 (80.9–80.9)	22.5	68.0
*Stenotrophomonas pavanii* DSM 25135^T^	81.7	80.9	81.0	83.3 (83.0–82.9)	25.2	66.9
*Stenotrophomonas terrae* DSM 18941^T^	79.0	78.2	78.3	81.3 (80.9–80.9)	22.6	63.9
*Pseudomonas pictorum* JCM 9942^T^	80.0	79.1	79.8	81.8 (81.6–81.5)	23.0	66.0
*Pseudomonas geniculata* JCM 13324^T^	81.8	80.9	81.6	83.3 (82.9–82.8)	25.0	66.2
*Pseudomonas beteli* LMG 978^T^	81.8	81.0	81.6	83.3 (83.0–83.0)	25.0	66.8
*Pseudomonas hibiscicola* ATCC 19867^T^	81.6	80.9	81.5	83.2 (82.8–82.8)	24.8	66.4

*DDH estimate (identities/HSP length formula).

†Two-way ANI (One-way ANI 1–One-way ANI 2).

Based on DNA-based comparisons presented above, the four species of the genus *Stenotrophomonas* phylogenetically most closely related to BII-R7^T^ (*S. pavanii*, *S. maltophilia*, *S. rhizophila* and *S. chelatiphaga*) were compared with BII-R7^T^. The other distantly related remaining species of the genus *Stenotrophomonas* were not included in this comparative survey. Certain cultural, physiological, chemotaxonomic and biochemical key features of these strains of members of the genus *Stenotrophomonas* were analyzed, even if these analyses had been performed in previous studies, in order to guarantee a comprehensive comparative study.

The morphology of cells grown on LB broth at 28 °C for 24 h with shaking at 160 r.p.m. was observed by scanning electron microscopy (Quanta 400, FEI; Fig. S1). Gram staining, cell motility and the presence of flagella were determined according to the method of Komagata [43]. At the physiological level, catalase activity was determined by assessing bubble production in 3 % (v/v) H_2_O_2_, and oxidase activity by using a 1 % (w/v) solution of tetramethyl-*p*-phenylenediamine [44]. The growth capacity at various temperatures (4, 15, 20, 28, 37 and 40 °C), NaCl concentrations (0, 0.5, 1, 1.5, 2.5 and 5 % at 28 °C) and pH values (pH 4.0–13.0 using increments of 1.0 pH units at 28 °C) was determined in TSB culture medium, except in the case of the 0 % NaCl test, which was performed in R2A culture medium. Anaerobic growth was not detected when BII-R7^T^was cultivated in serum bottles containing R2A broth, supplemented with thioglycolate (1 g l^−1^) and the upper gas phase replaced with nitrogen. However, the ability to reduce nitrate indicates that anaerobic growth might occur under certain circumstances. Carbon sources utilization, acid production from carbon sources and some physiological characteristics were determined by using the API 20NE (48 h, 28 °C), API 50CH (inoculated with AUX medium, 48 h, 28 °C) and API ZYM (4 h, 28 °C) galleries, respectively, according to the instructions of the manufacturer (bioMérieux) and the methods of Kämpfer *et al.* [45]. Some of these cultural and physiological characteristics of BII-R7^T^, including carbon source utilization and acid formation from these carbon sources, were compared with those of the reference strains (Table 2), and some differences were detected.

**Table 2.** Differential phenotypic characteristics between *Stenotrophomonas bentonitica* sp. nov. and the phylogenetically closest species of the genus *Stenotrophomonas* with validly published names Strains: 1, BII-R7^T^; 2, *S. rhizophila* DSM 14405^T^; 3, *S. pavanii* DSM 25135^T^; 4, *S. maltophilia* DSM 50170^T^; 5, *S. chelatiphaga* DSM 21508^T^. All data from this study. All strains were positive for catalase and protease (gelatin hydrolysis) activity and for acid formation from d-glucose, d-mannose and maltose. All strains were negative for acid formation from lactose, d-mannitol, dulcitol, adonitol, inositol, sorbitol, l-arabinose, raffinose, l-rhamnose, d-xylose, cellobiose, methyl d-glucoside, melibiose and d-arabitol. All strains hydrolysed: aesculin, oNP-β-d-galactopyranoside, pNP-α-d-glucopyranoside, pNP-β-d-glucopyranoside, Bis-pNP-phosphate, pNP-phenyl-phosphonate, pNP-phosphoryl-choline, l-alanine-pNA, l-glutamate-gamma-3-carboxy-pNA and l-proline-pNA but did not hydrolyse pNP-β-d-glucuronide. All strains utilised as sole sources of carbon: *N*-acetyl-d-galactosamine, *N*-acetyl-d-glucosamine, d-glucose, maltose, d-mannose, acetate, propionate, fumarate, dl-lactate, malate, pyruvate, d-ribose, salicin and trehalose. None of the tested strains utilised: l-arabinose, d-galactose, d-gluconate, l-rhamnose, d-adonitol, d-inositol, d-mannitol, sorbitol, putrescine, adipate, 4-aminobutyrate, azelate, dl-3-hydroxybutyrate, itaconate, mesaconate, oxoglutarate, suberate, β-alanine, l-aspartate, l-leucine, l-phenylalanine, l-serine, l-tryptophan, 3-hydroxybenzoate, 4-hydroxybenzoate or phenylacetate. +, Positive; −, negative; w, weakly positive.

Characteristic	1	2	3	4	5
Growth at/with:					
4 °C	−	+	−	−	−
40 °C	−	−	−	−	+
pH 12	−	−	+	−	−
5 % NaCl	−	−	−	+	−
Motility	−	−	−	+	+
Indole production	−	−	−	−	+
Nitrate reduction to nitrite	+	+	−	+	−
Hydrolysis of:					
pNP-β-d-xylopyranoside	+	+	+	−	−
Enzyme activity:					
Oxidase	−	+	−	−	+
Urease	−	−	−	+	−
β-Galactosidase	w	w	+	w	+
Utilization of:					
d-Fructose	−	−	+	+	−
Sucrose	−	+	+	+	−
d-Xylose	+	+	+	−	+
Maltitol	−	+	−	+	−
l-Histidine	−	−	+	+	+
Cellobiose	+	−	+	+	+
Glutarate	+	−	−	−	−
Melibiose	+	+	+	−	−
*cis*-Aconitate	−	−	+	+	−
*trans*-Aconitate	−	−	+	+	−
l-Alanine	−	−	+	+	+
l-Ornithine	−	−	−	+	−
l-Proline	−	−	+	+	+
*p*-Arbutin	+	−	+	+	−
Acid formation from:					
d-Galactose	−	−	−	−	+
Salicin	−	+	+	−	−

Biomasses subjected to extraction of polyamines, quinones and polar lipids were grown in PYE broth [0.3 % peptone from casein (w/v), 0.3 % yeast extract (w/v), pH 7.2] at 28 °C. Polyamines were extracted from biomasses harvested at the late exponential growth phase according to the protocol of Busse and Auling [46]. HPLC equipment was described by Stolz *et al.* [47] and conditions for HPLC analysis were described by Busse *et al*. [48]. The polyamine pattern of BII-R7^T^ consisted of the major polyamine spermidine [90.0 µmol (g dry weight)^−1^], moderate amounts of spermine [8.5 µmol (g dry weight)^−1^] and traces [<0.2 µmol (g dry weight)^−1^] of cadaverine, putrescine and 1,3-diaminopropane. This polyamine pattern was very similar to that of *S. chelatiphaga* DSM 21508^T^ which has also been found to contain spermidine as the major polyamine [78.8 µmol (g dry weight)^−1^], moderate amounts of spermine [6.2 µmol (g dry weight)^−1^] and traces [<0.2 µmol (g dry weight)^−1^] of cadaverine, putrescine and 1,3-diaminopropane. On the other hand, the absence of significant amounts of cadaverine distinguish BII-R7^T^ and *S. chelatiphaga* DSM 21508^T^ from other species of the genus *Stenotrophomonas*. *S. rhizophila* DSM 14405^T^ was found to have a polyamine pattern consisting of the major polyamines spermidine [87.1 µmol (g dry weight)^−1^] and cadaverine [17.5 µmol (g dry weight)^−1^] and moderate amounts of spermine [4.5 µmol (g dry weight)^−1^] and the polyamine pattern of *S. pavanii* DSM 25135^T^ contained the major polyamines spermidine [58.3 µmol (g dry weight)^−1^] and cadaverine [16.3 µmol (g dry weight)^−1^], small amounts of spermine [2.3 µmol (g dry weight)^−1^] and traces [<0.2 µmol (g dry weight)^−1^] of putrescine. The polyamine pattern with high amounts of cadaverine resembles that of *S. maltophilia* which has been reported to show a pattern with almost equal amounts of cadaverine and spermidine [49].

Quinones and polar lipids were extracted from biomass harvested at the stationary growth phase applying the integrated protocol of Tindall [50, 51] and Altenburger *et al*. [52]. The HPLC equipment used for quinone analysis has been described recently [47]. The quinone system consisted of ubiquinone Q-8 (98.8 %) and Q-7 (1.2 %). The polar lipid profiles (Fig. S2) of BII-R7^T^ and the reference species showed the presence of the major lipid diphosphatidylglycerol, moderate amounts of phosphatidylglycerol and phosphatidylethanolamine and minor amounts of the unidentified aminophospholipid APL1. In the case of BII-R7^T^ and *S. chelatiphaga* DSM 21508^T^ their polar lipid profiles were almost identical, showing only some small quantitative differences in the lipids detected. Also, *S. rhizophila* DSM 14405^T^ was highly similar but some minor lipids were not detected in this species (phospholipid PL1, and the two lipids L2 and L3, only visible after detecting total lipids). *S. pavanii* DSM 25135^T^ was distinguishable from BII-R7^T^on the basis of the presence of unidentified lipids including glycolipid GL1, aminolipid AL1, lipid L4 and phospholipid PL2. *S. maltophilia* DSM 50170^T^ could be distinguished from BII-R7^T^ by the presence of the unidentified lipids aminolipid AL2 and the two lipids L5 and L6 and absence of lipids L1, L2 and L3.

Biomass for fatty acid analysis was harvested after growth on TSA at 28 °C for 48 h. The analysis was performed as described by Kämpfer and Kroppenstedt [53]. Fatty acids were separated with a 5898A gas chromatograph (Hewlett Packard), the respective peaks were automatically integrated and fatty acid names and percentages were determined with the Sherlock MIDI software version2.1 (TSBA v. 4.1). The fatty acid profile of BII-R7^T^ was consistent with the profiles described for species of the genus *Stenotrophomonas* as shown in Table 3, with the predominant unsaturated fatty acids iso-C_15 : 0_ and anteiso-C_15 : 0_ and a variety of iso-branched hydroxylated fatty acids, typical of representatives of the genus *Stenotrophomonas* (Table 3).

**Table 3.** Fatty acid compositions of BII-R7^T^ and other members of the genus *Stenotrophomonas* Strains: 1, BII-R7^T^; 2, *S. rhizophila* DSM 14405^T^; 3, *S. pavanii* DSM 25135^T^; 4, *S. maltophilia* DSM 50170^T^; 5, *S. chelatiphaga* DSM 21508^T^. All data are from this study. Strains were grown on TSA at 28 °C for 48 prior to analysis. −, Not detected.

Fatty acid	1	2	3	4	5
C_10 : 0_	–	0.6	0.6	0.9	2.0
iso-C_11 : 0_	3.7	3.0	3.3	3.0	4.2
Unknown ECL* 11.799	1.0	0.7	1.3	1.5	1.6
iso-C_11 : 0_ 3-OH	2.0	1.6	1.4	1.8	2.9
iso-C_13 : 0_	–	–	–	–	2.2
anteiso-C_13 : 0_	–	–	–	–	2.0
iso-C_12 : 0_ 3-OH	–	0.4	–	–	1.1
C_12 : 0_ 3-OH	3.6	2.0	1.6	4.1	4.3
iso-C_14 : 0_	1.4	1.0	0.8	–	4.0
C_14 : 0_	2.3	1.0	1.9	2.5	15.8
iso-C_13 : 0_ 3-OH	3.2	1.8	3.4	3.3	1.9
C_13 : 0_ 2-OH	1.7	0.9	0.9	–	1.6
iso-C_15 : 1_	1.1	1.6	–	–	6.3
iso-C_15 : 0_	23.8	17.9	30.1	29.4	10.6
anteiso-C_15 : 0_	19.4	22.2	23.3	13.3	10.8
C_15 : 0_	0.9	0.7	–	–	1.4
iso-C_16 : 0_	4.0	3.7	1.9	–	1.1
C_16 : 1_ω9*c*	2.3	3.2	2.2	4.2	3.9
Summed feature 3†	9.2	8.4	6.1	8.3	13.7
C_16 : 0_	9.4	8.6	7.0	13.7	7.4
iso-C_17 : 1_ω9*c*	7.5	10.0	4.0	3.9	1.5
iso-C_17 : 0_	2.8	4.8	4.2	4.7	–
anteiso-C_17 : 0_	–	0.9	1.0	–	–
C_17 : 1_ω8*c*	–	0.7	–	–	–
cyclo-C_17 : 0_	1.7	1.6	2.4	–	–
C_18 : 1_ω9*c*	–	1.0	1.7	2.2	–
C_18 : 1_ω7*c*	–	1.4	0.9	1.7	–

*ECL, equivalent chain length.

†Summed feature 3: C_16 : 1_ω7*c*/ikso-C_15__ : 0_ 2-OH.

BII-R7^T^ shows molecular and phenotypic characteristics typical of the members of the genus *Stenotrophomonas* while it can be clearly differentiated from other members of this genus by a number of significant characteristics. At the molecular level, the results of the 16S rRNA and *gyrB* phylogenetic analyses and the different genome-based indices calculated confirmed unequivocally the status of BII-R7^T^ as representing a novel species and that *S. rhizophila*, *S. maltophilia*, *S. pavanii* and *S. chelatiphaga* are the most closely related species within the genus. Besides these differences at the DNA level, these four most closely related species of the genus *Stenotrophomonas* can be distinguished with regard to several phenotypic features (Table 2). Although results of polar lipid and polyamine analyses showed high similarity between BII-R7^T^ and *S. chelatiphaga* DSM 21508^T^, these strains presented contrasting results with respect to other aspects such as fatty acid composition (Table 3), motility, indole production capacity and certain enzymatic activities like oxidase (Table 2). In the case of the recently described novel species of the genus *Stenotrophomonas*, *S. tumulicola*, a comprehensive comparison was performed at different levels based on the data obtained for BII-R7^T^ in this study and the data extracted from the publication of Handa *et al.* [24]. 16S rRNA and *gyrB* gene sequence differences (Tables S1–S3) were supported by a number of phenotypic differences, such as growth inhibition in the presence of 5 % NaCl, lack of cell motility and ability to reduce nitrates to nitrites.

On this basis, BII-R7^T^ represents a novel species of the genus *Stenotrophomonas*, for which the name *Stenotrophomonas bentonitica* sp. nov. is proposed.

## Description of *Stenotrophomonas bentonitica* sp. nov.

*Stenotrophomonas bentonitica* (ben.to.ni′ti.ca. N.L. fem. adj. *bentonitica* referring to bentonite, the type of clay from which this bacterium was isolated).

Cells are Gram-stain negative, aerobic, non-motile, with no flagella and do not form endospores. When grown on LB agar at 28 °C for 3 days, colonies are light yellow, smooth, convex and circular. Under these growing conditions, cells are straight rods 0.45–0.65 µm in width and 0.92–1.55 µm in length and occur singly or in pairs. Growth takes place at 15–37 °C but not at 4 or 40 °C (optimum is 28 °C) at pH 5–10 but not at pH 4 or 11 (optimum at pH 7) and with 0–2.5 % NaCl but not with 5 % NaCl. The organism is able to reduce nitrate to nitrite but not to N_2_. Catalase, leucine arylamidase, protease, esterase, esterase lipase, acid and alkaline phosphatase, naphtol-AS-BI-phosphohydrolase and β-glucosidase activities are positive but oxidase activity is negative. No indole production occurs. The strain is able to utilize the following carbon sources: d-xylose, cellobiose, glutarate, melibiose, *p*-arbutin, *N*-acetyl-d-galactosamine, *N*-acetyl-d-glucosamine, d-glucose, maltose, d-mannose, acetate, propionate, fumarate, dl-lactate, malate, pyruvate, d-ribose, salicin and trehalose. The predominant fatty acids are iso-C_15 : 0_ and anteiso-C_15 : 0_. In addition a variety of iso-branched hydroxylated fatty acids are produced. The polyamine pattern contains the major polyamine spermidine, moderate amounts of spermine and traces of cadaverine, putrescine and 1,3-diaminopropane. The quinone system is composed of the major compound ubiquinone Q-8 and small amounts of Q-7. The polar lipid profile contains the major lipid diphosphatidylglycerol, moderate amounts of phosphatidylglycerol, and phosphatidylethanolamine and minor amounts of an unidentified aminophospholipid, a phospholipid and three lipids.

The type strain is *Stenotrophomonas bentonitica* BII-R7^T^ (=LMG 29893^T^=CECT 9180^T^=DSM 103927^T^), and was isolated from bentonite formations. The DNA G+C content of the type strain is 66.5 mol%.

## Supplementary Data

2885 Supplementary File 1
