# Supplementary material for: Stenotrophomonas bentonitica sp. nov., isolated from bentonite formations
Source: Int J Syst Evol Microbiol. 2017 Aug 18;67(8):2779–86. doi: 10.1099/ijsem.0.002016 (PMC5817250; doi:10.1099/ijsem.0.002016)
Supplement: Supplementary File 1 [file ijsem-67-2779-s001.pdf]

Article title: ***Stenotrophomonas bentonitica* sp. nov., isolated from bentonite formations**

Author names: Iván Sánchez-Castro[1,*], Miguel A. Ruiz-Fresneda[1], Mohammed Bakkali[2], Peter Kämpfer[3], Stefanie P. Glaeser[3], Hans-Jürgen Busse[4], Margarita López-Fernández[1]§, Pablo Martínez-Rodríguez[1], Mohamed L. Merroun[1]

Journal name: International Journal of Systematic and Evolutionary Microbiology

Corresponding author: Iván Sánchez-Castro, Departamento de Microbiología, Campus de Fuentenueva, Universidad de Granada, 18071 Granada, Spain – Email: ivansanchezcastro@gmail.com – Phone number: +34958249331

Table S1. Pairwise 16S rRNA gene sequences similarities of strain BII-R7^T to type strains of all *Stenotrophomonas* species as well as wrongly classified *Pseudomonas* species. Pairwise 16S rRNA gene sequence similarities were determined in ARB using the ARB Neighbour-joining tool without applying an evolutionary model for sequence similarity calculations. Red highest (100%) to green lowers 16S rRNA gene sequence similarities.

	***S. bentonitica* BII-R7^T (HG800055)**	*S. rhizophila* e-p10^T (AJ293463)	*S. panacihumi* MK06^T (GQ856217)	*S. maltophilia* IAM 12423^T (AB294553)	*S. pavanii* ICB 89^T (FJ748683)	[*P. beteli*] ATCC 19861^T (AB021406)	[*P. geniculata*] ATCC 19374^T (AB021404)	[*P. hibiscicola*] ATCC 19867^T (AB021405)	*S. chelatiphaga* LPM-5^T (EU573216)	*S. tumulicola* T5916-2-1b^T (LC066089)	*S. ginsengisoli* DCY01^T (DQ109037)	*S. koreensis* TR6-01^T (AB166885)	[*P. pictorum*] LMG 981^T (AJ131116)	*S. terrae* R-32768^T (AM403589)	*S. nitritireducens* L2^T (AJ012229)	*S. humi* R-32729^T (AM403587)	*S. acidaminiphila* AMX19^T (AF273080)	*S. onas daejeonensis* MJ03^T (GQ241320)
***Stenotrophomonas bentonitica* BII-R7^T (HG800055)**	100	99.2	97.9	98.3	98.5	97.8	97.9	97.7	98.3	98.3	97	97.2	97.3	97.3	97.4	97.8	96.8	96.6
Stenotrophomonas rhizophila e-p10^T (AJ293463)	99.2	100	98.7	97.9	98.1	97.5	97.9	97.7	98.3	98.4	96.5	97.1	97.3	97.4	97.3	97.6	96.9	96.3
Stenotrophmonas panacihumi MK06^T (GQ856217)	97.9	98.7	100	97.4	97.6	97	97.3	97.1	97.7	97.8	96	96.4	96.4	96.6	96.6	97	96.3	96.1
Stenotrophomonas maltophilia IAM 12423^T (AB294553)	98.3	97.9	97.4	100	99.7	99.4	99.4	99.5	98.6	98.6	96.6	97.2	97.1	97.4	97.5	97.5	97.2	97
Stenotrophomonas pavanii ICB 89^T (FJ748683)	98.5	98.1	97.6	99.7	100	99.1	99.3	99.2	98.6	98.7	96.7	97.3	97.1	97.4	97.5	97.6	97.2	97
[*Pseudomonas beteli*] ATCC 19861^T (AB021406)	97.8	97.5	97	99.4	99.1	100	99.2	99.3	98.2	98.3	96.7	97	96.6	97	97.2	97	97.1	96.9
[*Pseudomonas geniculata*] ATCC 19374^T (AB021404)	97.9	97.9	97.3	99.4	99.3	99.2	100	99.5	98.6	98.7	96.8	97.7	97	97.4	97.2	97.2	97.2	96.5
[*Pseudomonas hibiscicola*] ATCC 19867^T (AB021405)	97.7	97.7	97.1	99.5	99.2	99.3	99.5	100	98.4	98.5	96.9	97.3	96.8	97.1	97	97	97	96.6
Stenotrophomonas chelatiphaga LPM-5^T (EU573216)	98.3	98.3	97.7	98.6	98.6	98.2	98.6	98.4	100	99.4	97.4	97.6	97.1	97.4	97.2	97.4	97	97
Stenotrophimonas tumulicola T5916-2-1b^T (LC066089)	98.3	98.4	97.8	98.6	98.7	98.3	98.7	98.5	99.4	100	97.1	97.7	97.4	97.6	97.4	97.7	97.4	97
Stenotrophomonas ginsengisoli DCY01^T (DQ109037)	97	96.5	96	96.6	96.7	96.7	96.8	96.9	97.4	97.1	100	98.2	96.7	96.8	96.5	97.1	96.3	96.6
Stenotrophomonas koreensis TR6-01^T (AB166885)	97.2	97.1	96.4	97.2	97.3	97	97.7	97.3	97.6	97.7	98.2	100	96.9	97.5	97.3	97.1	97.5	96.5
[*Pseudomonas pictorum*] LMG 981^T (AJ131116)	97.3	97.3	96.4	97.1	97.1	96.6	97	96.8	97.1	97.4	96.7	96.9	100	98.8	98.7	98.7	98.1	96.9
Stenotrophomonas terrae R-32768^T (AM403589)	97.3	97.4	96.6	97.4	97.4	97	97.4	97.1	97.4	97.6	96.8	97.5	98.8	100	99.3	99	98.6	97.1
Stenotrophomonas nitritireducens L2^T (AJ012229)	97.4	97.3	96.6	97.5	97.5	97.2	97.2	97	97.2	97.4	96.5	97.3	98.7	99.3	100	99.1	98.5	97.4
Stenotrophomonas humi R-32729^T (AM403587)	97.8	97.6	97	97.5	97.6	97	97.2	97	97.4	97.7	97.1	97.1	98.7	99	99.1	100	98	97.4
Stenotrophomonas acidaminiphila AMX19^T (AF273080)	96.8	96.9	96.3	97.2	97.2	97.1	97.2	97	97	97.4	96.3	97.5	98.1	98.6	98.5	98	100	98.3
Stenotrophomonas daejeonensis MJ03^T (GQ241320)	96.6	96.3	96.1	97	97	96.9	96.5	96.6	97	97	96.6	96.5	96.9	97.1	97.4	97.4	98.3	100

Table S2. Pairwise *gyrB* gene (region 1) sequences similarities of strain BII-R7^{T} and type strains of species of the genus *Stenotrophomonas* as well as the wrongly-classified strain *Pseudomonas beteli*. Pairwise *gyrB* gene sequence similarities were calculated based on *p*-distances in MEGA 7. Red highest (100%) to green lowers gyrB gene sequence similarities.

	S. bentonitica BII-R7^{T} (MKCZ00000000)	*S. chelatiphaga* CCUG 57178^{T} (GU945517)	*S. daejeonensis* CCUG 59871^{T} (HQ434503)	*S. rhizophila* CCUG 54934^{T} (GU945512)	*S. pavanii* CCUG 59972^{T} (HQ434505)	[*P. beteli*] LMG 978^{T} (HQ434506)	S *S. ginsengisoli* CCUG 59870^{T} (HQ434502)	*S. nitritireducens* CCUG 46888^{T} (GU945508)	*S. acidaminiphila* CCUG 46887^{T} (GU945510)	*S. maltophilia* CCUG 5866^{T} (GU945506)	*S. terrae* CCUG 54880^{T} (GU945516)	S *S. panacihumi* CCUG 59872^{T} (HQ434504)	*S. humi* CCUG 54881^{T} (GU945515)	*S. tumulicola* T5916-2-1b^{T} (LC092950)	*S. koreensis* CCUG 53887^{T} (GU945514)
S. bentonitica BII-R7^{T} (MKCZ00000000)	100.0	89.9	89.3	89.1	88.2	88.1	87.7	87.6	87.3	87.1	86.7	86.4	86.2	83.3	83.0
S. chelatiphaga CCUG 57178^{T} (GU945517)	89.9	100.0	88.3	89.0	87.7	88.1	87.3	87.5	86.9	87.6	85.9	86.4	88.6	83.1	83.0
S. daejeonensis CCUG 59871^{T} (HQ434503)	89.3	88.3	100.0	90.4	90.5	90.0	87.8	92.2	92.1	89.5	88.4	90.0	90.4	84.2	85.5
S. rhizophila CCUG 54934^{T} (GU945512)	89.1	89.0	90.4	100.0	89.3	89.2	87.4	89.0	87.6	88.3	87.0	87.5	89.0	83.7	85.5
S. pavanii CCUG 59972^{T} (HQ434505)	88.2	87.7	90.5	89.3	100.0	96.6	88.2	89.8	89.5	95.4	85.2	88.4	88.9	82.2	85.1
[*P. beteli*] LMG 978^{T} (HQ434506)	88.1	88.1	90.0	89.2	96.6	100.0	88.4	89.9	89.5	94.8	85.4	88.2	88.9	83.1	85.3
S. ginsengisoli CCUG 59870^{T} (HQ434502)	87.7	87.3	87.8	87.4	88.2	88.4	100.0	88.1	88.2	88.5	85.1	86.7	86.7	82.2	85.4
S. nitritireducens CCUG 46888^{T} (GU945508)	87.6	87.5	92.2	89.0	89.8	89.9	88.1	100.0	92.9	89.5	87.7	89.7	88.5	83.0	86.2
S. acidaminiphila CCUG 46887^{T} (GU945510)	87.3	86.9	92.1	87.6	89.5	89.5	88.2	92.9	100.0	89.7	86.2	89.5	88.6	83.2	85.9
S. maltophilia CCUG 5866^{T} (GU945506)	87.1	87.6	89.5	88.3	95.4	94.8	88.5	89.5	89.7	100.0	84.6	88.1	88.6	82.7	86.4
S. terrae CCUG 54880^{T} (GU945516)	86.7	85.9	88.4	87.0	85.2	85.4	85.1	87.7	86.2	84.6	100.0	86.0	87.4	84.4	81.5
S. panacihumi CCUG 59872^{T} (HQ434504)	86.4	86.4	90.0	87.5	88.4	88.2	86.7	89.7	89.5	88.1	86.0	100.0	87.5	81.6	85.5
S. humi CCUG 54881^{T} (GU945515)	86.2	88.6	90.4	89.0	88.9	88.9	86.7	88.5	88.6	88.6	87.4	87.5	100.0	84.9	86.3
S. tumulicola T5916-2-1b^{T} (LC092950)	83.3	83.1	84.2	83.7	82.2	83.1	82.2	83.0	83.2	82.7	84.4	81.6	84.9	100.0	80.5
S. koreensis CCUG 53887^{T} (GU945514)	83.0	83.0	85.5	85.5	85.1	85.3	85.4	86.2	85.9	86.4	81.5	85.5	86.3	80.5	100.0

Table S3. Pairwise *gyrB* gene (region 2) sequences similarities of strain BII-R7^{T} and type strains of species of the genus *Stenotrophomonas* as well as the wrongly-classified strain *Pseudomonas beteli*. Pairwise *gyrB* gene sequence similarities were calculated based on *p*-distances in MEGA 7. Red highest (100%) to green lowers gyrB gene sequence similarities.

	S. bentonitica BII-R7^{T} (MKCZ00000000)	*S. rhizophila* CCUG 54934^{T} (GU945526)	*S. acidaminiphila* CCUG 46887^{T} (GU945524)	*S. pavanii* CCUG 59972^{T} (HQ434521)	*S. daejeonensis* CCUG 59871^{T} (HQ434519)	*S. nitritireducens* CCUG 46888^{T} (GU945522)	[*P. beteli*] LMG 978^{T} (HQ434522)	*S. maltophilia* CCUG 5866^{T} (GU945520)	S. humi CCUG 54881T (GU945529)	*S. tumulicola* T5916-2-1b^{T} (LC092951)	*S. terrae* CCUG 54880^{T} (GU945530)	*S. chelatiphaga* CCUG 57178^{T} (GU945531)	*S. panacihumi* CCUG 59872^{T} (HQ434520)	*S. ginsengisoli* CCUG 59870^{T} (HQ434518)	*S. koreensis* CCUG 53887^{T} (GU945528)
S. bentonitica BII-R7^{T} (MKCZ00000000)	100.0	87.2	84.4	84.3	83.9	83.7	82.6	82.5	81.9	81.5	81.5	78.3	77.7	73.5	72.5
S. rhizophila CCUG 54934^{T} (GU945526)	87.2	100.0	83.6	82.9	82.9	82.5	82.6	82.7	80.9	80.5	80.4	78.5	76.0	73.6	72.8
S. acidaminiphila CCUG 46887^{T} (GU945524)	84.4	83.6	100.0	83.3	91.4	92.1	82.2	82.6	86.8	82.3	87.1	82.2	78.8	74.6	74.6
S. pavanii CCUG 59972^{T} (HQ434521)	84.3	82.9	83.3	100.0	84.4	82.9	93.4	93.8	82.2	82.0	80.2	82.2	76.9	75.5	74.5
S. daejeonensis CCUG 59871^{T} (HQ434519)	83.9	82.9	91.4	84.4	100.0	92.3	83.3	83.6	86.8	82.2	87.5	82.3	80.8	75.0	74.5
S. nitritireducens CCUG 46888^{T} (GU945522)	83.7	82.5	92.1	82.9	92.3	100.0	83.9	82.7	86.5	81.9	87.4	82.6	78.8	75.5	74.9
[*P. beteli*] LMG 978^{T} (HQ434522)	82.6	82.6	82.2	93.4	83.3	83.9	100.0	93.8	83.2	83.2	80.4	83.3	76.2	73.9	72.9
S. maltophilia CCUG 5866^{T} (GU945520)	82.5	82.7	82.6	93.8	83.6	82.7	93.8	100.0	82.9	83.2	79.8	83.0	75.9	74.3	74.1
S. humi CCUG 54881^{T} (GU945529)	81.9	80.9	86.8	82.2	86.8	86.5	83.2	82.9	100.0	80.2	85.8	81.8	75.3	74.5	73.1
S. tumulicola T5916-2-1b^{T} (LC092951)	81.5	80.5	82.3	82.0	82.2	81.9	83.2	83.2	80.2	100.0	78.5	83.9	74.8	75.0	72.9
S. terrae CCUG 54880^{T} (GU945530)	81.5	80.4	87.1	80.2	87.5	87.4	80.4	79.8	85.8	78.5	100.0	79.4	76.7	73.2	72.4
S. chelatiphaga CCUG 57178^{T} (GU945531)	78.3	78.5	82.2	82.2	82.3	82.6	83.3	83.0	81.8	83.9	79.4	100.0	73.5	72.4	71.2
S. panacihumi CCUG 59872^{T} (HQ434520)	77.7	76.0	78.8	76.9	80.8	78.8	76.2	75.9	75.3	74.8	76.7	73.5	100.0	69.1	70.8
S. ginsengisoli CCUG 59870^{T} (HQ434518)	73.5	73.6	74.6	75.5	75.0	75.5	73.9	74.3	74.5	75.0	73.2	72.4	69.1	100.0	87.1
S. koreensis CCUG 53887^{T} (GU945528)	72.5	72.8	74.6	74.5	74.5	74.9	72.9	74.1	73.1	72.9	72.4	71.2	70.8	87.1	100.0

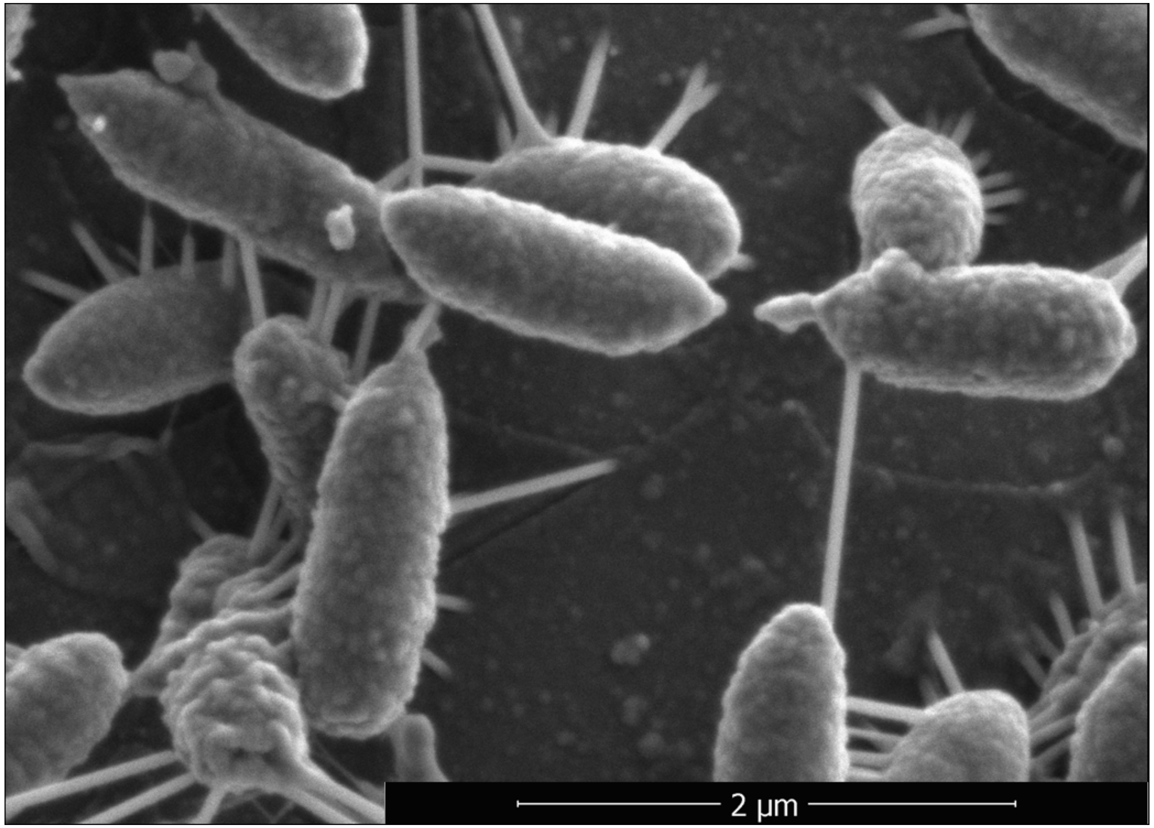

Figure S1. Scanning Electron Microscope (Quanta 400; FEI) image of BII-R7T cells grown on LB broth at 28 ºC for 24 h with shaking at 160 rpm.

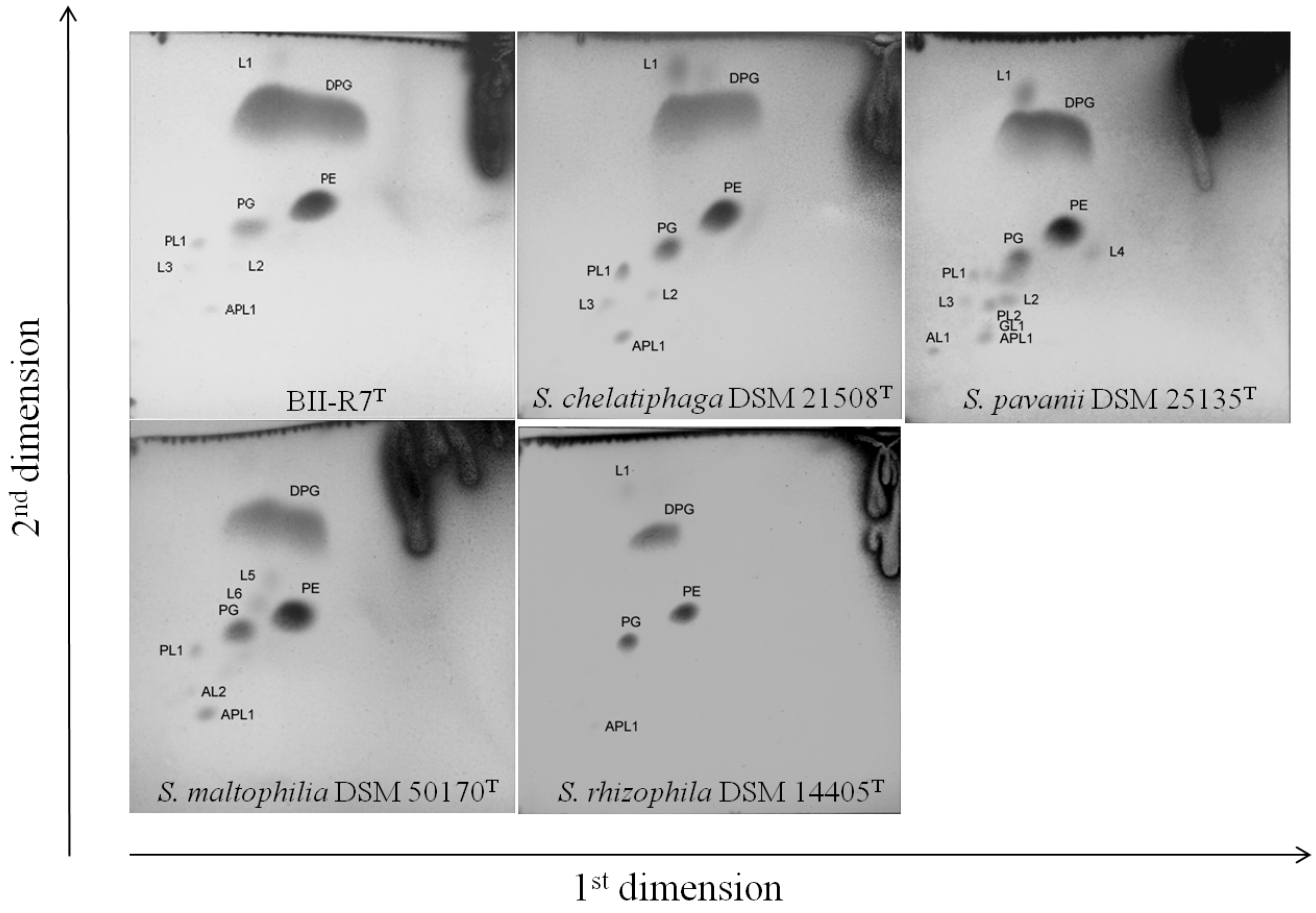

Figure S2. Polar lipid profiles of strain BII-R7^T and the species *S. pavanii* DSM 25135^T, *S. maltophilia* DSM 50170^T, *S. rhizophila* DSM 14405^T, *S. chelatiphaga* DSM 21508^T after two-dimensional thin-layer chromatography and visualized using ethanolic molybdatophosphoric acid. DPG, diphosphatidylglycerol; PG, phosphatidylglycerol; PE, phosphatidylethanolamine; GL1, unidentified glycolipid; APL1, unidentified aminophospholipid; PL1, PL2, unidentified phospholids; AL1, AL2, unidentified aminolipids; L1 – L6, unidentified polar lipid not reacting with the spray reagents specific for free amino groups, phosphate or sugar moieties.
